# Organoid Level Assessments of Human Primary and Metastatic Colorectal Cancer-Derived Organoids Predict Response to Chemotherapy and Chemoradiation

**DOI:** 10.3390/cancers18101587

**Published:** 2026-05-13

**Authors:** Shirsa Udgata, Alexa E. Schmitz, Amani A. Gillette, Alexandra G. Sorenson, Jeremiah M. Riendeau, Rian Engeldinger, Jordan N. Stoecker, Alyssa K. Steimle, Katherine A. Johnson, Devan Kittelson, Alexandra Isaak, Jeremy D. Kratz, Evie Carchman, Randall Kimple, Cheri A. Pasch, Melissa C. Skala, Dustin A. Deming

**Affiliations:** 1 Division of Hematology, Medical Oncology, and Palliative Care, Department of Medicine, University of Wisconsin School of Medicine and Public Health, University of Wisconsin-Madison, Madison, WI 53705, USA; 2 Morgridge Institute for Research, Madison, WI 53715, USA; 3 Department of Biomedical Engineering, University of Wisconsin-Madison, Madison, WI 53705, USA; 4 University of Wisconsin Carbone Cancer Center, Madison, WI 53705, USA; 5 School of Computer, Data, and Information Sciences, University of Wisconsin-Madison, Madison, WI 53705, USA; 6 Department of Surgery, William S. Middleton Memorial Veterans Hospital, Madison, WI 53705, USA; 7 Department of Surgery, University of Wisconsin School of Medicine and Public Health, University of Wisconsin-Madison, Madison, WI 53705, USA; 8 Department of Human Oncology, University of Wisconsin School of Medicine and Public Health, University of Wisconsin-Madison, Madison, WI 53705, USA; 9 McArdle Laboratory for Cancer Research, Department of Oncology, University of Wisconsin-Madison, Madison, WI 53705, USA

**Keywords:** patient-derived cancer organoids, colorectal cancer, preclinical cancer models, optical redox imaging, organoids

## Abstract

Background: Therapies for metastatic colorectal cancer (CRC) are largely chosen without considering inter-patient heterogeneity, leading to unnecessary side effects without clinical benefit for some patients. Patient-derived cancer organoids (PDCOs) faithfully recapitulate the morphology and molecular profiles of the primary tumors from which they are derived, making them attractive preclinical models for predicting response to standard therapies, and thus, for use in drug development assays. Methods: Here, we investigate the hidden subclonal driver mutations in CRC PDCOs and the importance of individual PDCO-level heterogeneity when addressing PDCO treatment responses using changes in diameter and optical redox imaging. PDCO response is then compared to clinical response across a cohort of subjects with CRC receiving chemotherapy and/or chemoradiation. Results: Change in diameter and optical redox imaging are more sensitive to detecting therapeutic response than endpoint and well-level measurements. These measurements accurately reflect clinical responses to chemotherapy and chemoradiation. Conclusions: Overall, these studies demonstrate the importance of PDCO-level methods of PDCO assessment and further establish PDCOs as powerful tools for drug response assessment and developmental therapeutic studies.

## Simple Summary

Patient-derived cancer organoids (PDCOs) have been established as faithful models of human disease, but the best methods for how to use these models to most accurately predict therapeutic response are still under development. Methods that measure changes in individual PDCOs over the course of treatment, such as change in individual organoid size over time and change in metabolic imaging, outperform methods that measure PDCOs at a single timepoint and at the whole-well level, and these measurements correlate with clinical outcomes in patients.

## 1. Introduction

The incidence and mortality of colorectal cancer (CRC) and locally advanced rectal cancer (LARC) have been steadily decreasing in patients over the age of 55 over the last 13 years [1]. However, since the 1990s, these rates have been increasing by 2% per year for patients under the age of 55 [1]. For patients with metastatic CRC, treatment is determined by the tumor’s pathological characteristics, molecular features, and the extent of disease [2], while patients with LARC typically receive total neoadjuvant therapy consisting of systemic chemotherapy (5-fluorouracil (5FU) and oxaliplatin containing regimens) and chemoradiation [3]. These varying clinical features, among others, lead to significant inter-patient heterogeneity in treatment success rates across the many treatment options available [4]. This heterogeneity results in some patients being over-treated, leading to unnecessary side effects and/or therapeutic resistance requiring further therapy escalation [4,5,6]. Hence, there remains an unmet need for reliable ex vivo models to predict patient response to clinical treatments and to reliably aid in the development of novel treatment strategies.

Patient-derived cancer organoids (PDCOs) have become the preferred models for drug response studies due to their ability to recapitulate the genomic changes in patients’ tumors better than two-dimensional (2D) cell cultures [4,7]. Previous studies from our laboratory showed that the considerable heterogeneity found within a PDCO culture can alter therapeutic sensitivity [4,7]. However, the current common methods to assess drug response for PDCOs do not consider single PDCO-level heterogeneity and do not allow longitudinal studies to track response to treatments [8,9,10,11]. A major hallmark of cancer cells is their altered metabolism, which compensates for their increased need for energy and nutrients [4,12,13]. A nondestructive way to measure metabolic changes in the cell is through label-free autofluorescence of metabolic cofactors, NADH, NADPH, and FAD [13,14]. NADH and NADPH have overlapping spectral properties and are jointly referred to as NAD(P)H [15]. The optical redox ratio (ORR) takes the autofluorescence intensity of NAD(P)H over the sum of NAD(P)H and FAD to determine the oxidation-reduction state of a cell at that given time [16]. The ORR increases with increasing level of glycolytic metabolism and decreases due to metabolic changes caused by drug treatments, specifically chemotherapy [14,17,18]. Previous work in our lab has shown that response to chemotherapy in PDCOs causes a decrease in diameter and ORR because of cell death and metabolic changes [4,5,13]. Here, we use the change in diameter and the optical redox imaging of individual PDCOs over time to assess PDCO response.

Additionally, prior studies from our lab have also shown that PDCOs reliably predicted response to various standard-of-care therapies across twenty different advanced solid cancers [4]. Here, we further investigate the presence of subclonal heterogeneity in PDCOs and the use of PDCO-level changes in diameter and metabolism to predict clinical patient response.

## 2. Materials and Methods

### 2.1. Patient Cancer Samples and PDCO Processing

All tissue sections from CRC and LARC patients were obtained with written informed consent according to the Institutional Review Board (IRB) approval through the University of Wisconsin (UW) Molecular Tumor Board Registry (UW IRB#2014-1370; approved 4 January 2016), the UW Translational Science BioCore (UW IRB#2016-0934), and the UW Rectal Cancer Registry (UW IRB#2018-0149; approved 17 April 2018). Tissue samples were obtained from needle biopsies, endoscopic biopsies, or primary surgical resection and placed in chelation buffer for at least 30 min. Further tissue processing was done as previously described [4,5,7]. PDCOs were plated in 50 µL droplets of a 1:1 ratio of Matrigel (Corning; Corning, NY, USA; cat #:354234) and Advanced DMEM/F12 containing HEPES, Glutamax, and pen strep (ADF_Stock_). Organoid media containing ADF_Stock_, WNT3aL conditioned media, and recombinant EGF supplement (WNT_Feed_) were overlayed on top of solidified Matrigel droplets [5].

### 2.2. Next Generation DNA Sequencing of PDCOs

PDCOs were collected from 4 to 6 wells at ~80% confluency, pelleted at 1500 rpm for 5 min and washed once with 1x sterile PBS. The PBS was aspirated, and the cell pellets were stored at −80 °C until DNA isolation. DNA isolation, library preparation, and sequencing were done as described previously [4].

### 2.3. Variant Calling and Analysis for Tumors and PDCOs

Sequencing data from tumor samples was obtained via clinical sequencing, with PDCO mutation analysis completed as described previously [4].

### 2.4. Treating PDCOs with Chemotherapy and Radiation

PDCOs were plated in a 1:1 ratio of Matrigel and ADF_Stock_ in gridded glass dishes (Ibidi; cat #:81166; Fitchburg, WI, USA) for 72 h prior to baseline imaging and treatment with chemotherapy and radiation (XRT). Chemotherapy agents were acquired from the UW Carbone Cancer Center Pharmacy. PDCO treatments were determined based on the patient’s treatment in the clinic. PDCOs were treated at concentrations of 10 µM 5FU, 5 µM oxaliplatin, 1.5 nM SN38 (active metabolite of irinotecan; cat#: HY-13704; MedChemExpress; Monmouth Junction, NJ, USA), and 2 Gy of XRT. On Day 0, they were imaged and treated with chemotherapy and/or XRT. After 48 h, the chemotherapy was removed, and PDCOs were given fresh WNT_Feed_ for an additional 48 h. On Day 4, PDCOs were re-imaged and discarded.

### 2.5. 3D CellTiter-Glo Assay for PDCOs

After PDCOs were imaged on Day 4, 450 μL of 3D CellTiter-Glo (Promega; cat#: G9681; Madison, WI, USA) reagent was added to each well and pipetted to break down the Matrigel. Plates were covered, shaken for 5 min, and incubated for 25 min at room temperature. After incubation, luminescence was read for all plates.

### 2.6. Brightfield and Widefield Optical Redox Imaging (WF-ORI) Image Acquisition

Brightfield (BF) and WF autofluorescence images were taken on a Nikon Eclipse Ti (Nishioi, Shinagawa-ku, Tokyo, Japan) with a SOLA light engine using a 4× (0.13NA, air) objective and a Hamamatsu Flash4 digital CMOS camera (Shizuoka, Japan). PDCOs were imaged on Days 0 (pre-treatment) and 4 (post-treatment). BF images were used to assess a normalized change in diameter as a measure of PDCO growth. Widefield optical redox imaging (WF-ORI) autofluorescence images of the two metabolic co-enzymes NAD(P)H and FAD were used to assess any change in metabolism of PDCOs between Day 0 and Day 4 post-treatment. NAD(P)H was excited for 3 s using a 360/40 nm filter at 40% power (0.25 mJ/cm^2^), and emissions were collected with a 400 nm dichroic mirror and a standard 4′,6-diamidino-2-phenylindole (DAPI, 460/50 nm) filter. FAD was sequentially excited for 3.5 s using a 480/30 nm filter at 40% power (0.531 mJ/cm^2^) and emissions collected with a 505 nm dichroic mirror and a standard fluorescein isothiocyanate (FITC, 535/20 nm) filter. Five fields of view (FOVs) (image size = 3.34 mm × 3.34 mm; 2044 × 2048 pixels) were taken of each sample with three technical replicates per sample condition and light doses below levels shown to keep cells viable [19,20].

### 2.7. Two-Photon Optical Metabolic Imaging (OMI) Acquisition and Analysis

Briefly, NAD(P)H and FAD were excited at 750 and 890 nm, respectively, using a tunable ultrafast pulsed laser (Coherent, Inc.; Saxonburg, PA, USA), an inverted microscope (Nikon, Eclipse Ti), and a 20× water (1.0NA, Zeiss; Oberkochen, Germany) objective. Both NAD(P)H and FAD images were obtained for the same field of view (NAD(P)H with a 440/80 nm and FAD with a 550/100 nm emission filter, respectively). Fluorescence lifetime images were collected using time-correlated single photon counting electronics (SPC-150, Becker and Hickl, Berlin, Germany; and Time Tagger Ultra, Swabian Instruments, Stuttgart, Germany) and a GaAsP photomultiplier tube (H7422P-40, Hamamatsu). Images (512 × 512 pixels) were obtained using a pixel dwell time of 4.8μs over 60 s total integration time. A Fluoresbrite YG microsphere (Polysciences Inc.; Warrington, PA, USA) was imaged as a daily standard for fluorescence lifetime. The bead lifetime decay curves were fit to a single exponential decay, and the fluorescence lifetime was measured to be 2.1 ns (*n* = 7), which is consistent with published values [21]. The instrument response function was measured using a second-harmonic generation signal from urea crystals excited at 890 nm, with a full width at half-maximum of 240 ps for the B&H card and 310 ps for Swabian.

A custom script was used to create masks for PDCO 2P images. An in-house trained CellPose model was used to generate nuclear masks [22]. The script then dilated the nuclear masks by 5 pixels and tunneled the original nucleus from the dilated mask. The script showed each image in the Napari interface, where the user could modify masks if necessary. A custom Python(v3.10.12) script applied the mask to the .sdt file and summed the photon decays for all pixels in each cellular mask using SPCImage (v8.0, Becker & Hickl GmbH) [23]. The decay curves were fit on a per-cell level, using a bin size of 0, to a biexponential model convolved with the instrument response function, using an iterative parameter optimization to obtain the lowest sum of the squared differences between the model and data (weighted least squares algorithm). The decay curves were then integrated to generate the intensity of each fluorophore. Here, we only report the intensity values, and the lifetime parameters are not reported.

### 2.8. Statistical Analysis

ImageJ v1.51–1.54 (imagej.net/ij/) was used to quantify the longest diameter of each PDCO on BF images from Days 0 and 4. A relative change in diameter was determined using the formula:

$$Relative\;change\;in\;Diameter\;(\%)=\frac{{Diameter}_{Day4}-D{iameter}_{Day0}}{{Diameter}_{Day0}}\times 100$$

Widefield autofluorescence images were analyzed using a trained CellPose model for PDCO segmentation and leading-edge analysis to isolate the signal to the 20 pixel (32 mm) edge of the PDCO [21]. Each channel was then background normalized to account for day-to-day variation in the widefield system, and the background normalized intensities for NAD(P)H and FAD were used to calculate the optical redox ratio (ORR) for all PDCOs using the formula below, where I is the background normalized intensity [24]:

$$ORR=\frac{I_{NAD\left(P\right)H}}{I_{NAD\left(P\right)H}+I_{FAD}}$$

The 2P ORR was calculated similarly, without background normalization, on a per-cell level. The change in the optical redox ratio (ΔORR) was then calculated for each individual PDCO using formulas previously cited [13,24].

Medians and standard deviations were calculated for all diameter and optical redox ratio measurements across all replicates. A modified version of Glass’s delta (mGlass’s Δ) was calculated using:

$$mG\Delta =\frac{\left({\tilde{x}}_{control}-{\tilde{x}}_{treatment}\right)}{\sigma_{control}}$$

With $\tilde{x}$ being the median of the relative change in diameter or ΔORR, and σ being the standard deviation of the corresponding measurement [25,26]. To correlate with clinical data, diameter change cutoffs for mGlass’s Δ of FOLFOX- and FOLFIRI-treated lines were determined from a prior publication [4]. A diameter mGlass’s Δ of 1.25 or higher correlated with partial response, 1.24 to 0.76 correlated with stable disease, and 0.75 or lower correlated with progressive disease. For PDCO lines treated with 5FU + XRT, a diameter mGlass’s Δ of 1 or higher correlated with partial response, 0.99 to 0 correlated with stable disease, and 0 or lower correlated with progressive disease. Clinical response for each patient was determined based on the RECIST v1.1 guidelines [27]. Clinical imaging was performed per standard practice. Measurements were performed by a clinical oncologist (DAD) blinded to organoid results until after completion of imaging assessment. A mGlass’s Δ was used to quantify the drug response for ΔORR measurement based on prior work, with a threshold value of >1.5 equivalent to treatment sensitive, and <0.0 as treatment resistant for FOLFOX and FOLFIRI treated lines [13]. For XRT and the combination of XRT with 5FU, the mGlass’s Δ threshold > 1 was evaluated as treatment sensitive, and <0 was considered treatment resistant. These thresholds represent current guidelines based on the 13 LARC samples with patient data included in the current study.

## 3. Results

### 3.1. PDCOs Retain the Molecular Profile of the Patient’s Tumor from Which They Are Derived

**Figure 1:**
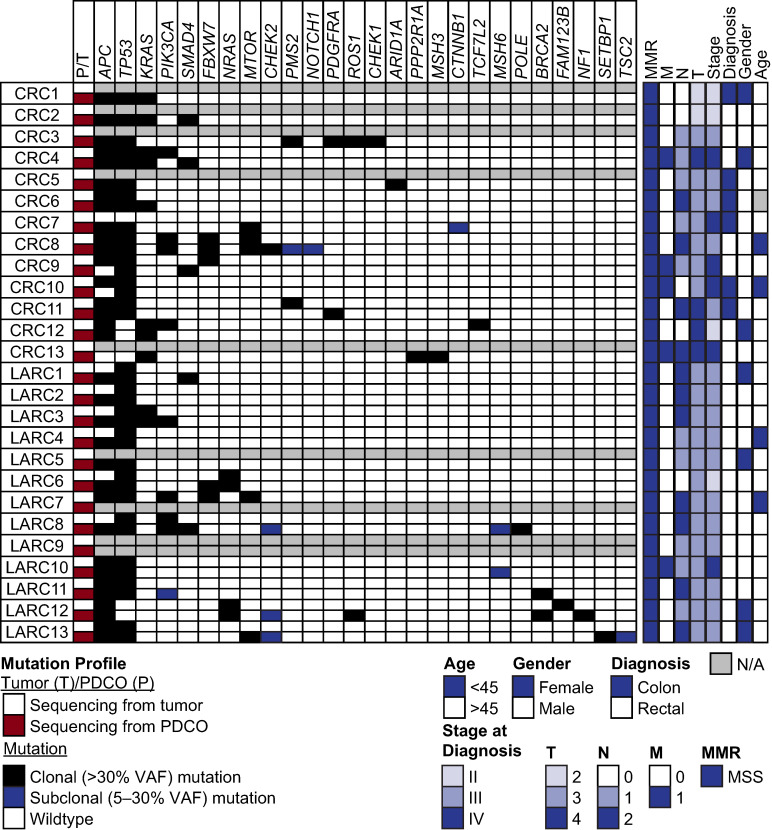
PDCOs retain the molecular profile of tumors from which they were derived. Clonal and subclonal mutations of all 26 PDCO lines included in this study. Black boxes indicate a clonal mutation (over 30% variant allele frequency (VAF)), blue boxes indicate a subclonal mutation (between 5 and 30% VAF), and white boxes indicate no variant was detected (wild type (WT)). Gray boxes indicate that sequencing was not performed on the line. For tumor and PDCO sequencing, red boxes indicate tumor sequencing, while white indicates sequencing from PDCOs. Patient demographics were determined for almost all patients. Gray boxes indicate no data available.

We have previously shown that PDCOs are representative models of the cancers from which they are derived [5]. To confirm this finding in a larger cohort and evaluate for the presence of unique alterations in the PDCO cultures, PDCO lines were generated from CRC and LARC patient cancer samples following consent on an IRB-approved protocol. The tissues were obtained following primary surgical resection pre- or post-treatment. Baseline demographics are presented in Figure 1.

DNA sequencing was performed for each PDCO line and compared to the patients’ clinical sequence data to confirm if the PDCOs retain the tumor molecular profile. PDCOs retained 76% of the driver mutations identified by tissue sequencing. Furthermore, 57% of PDCO samples had additional unique driver alterations not found in tissue sequencing. Unique subclonal variants (present at variant allele frequencies (VAFs) between 10 and 30%) not found in tissue sequencing were identified in 50% of samples (Figure 1).

### 3.2. PDCOs Show Differential Sensitivity to Chemotherapy and Radiation

**Figure 2:**
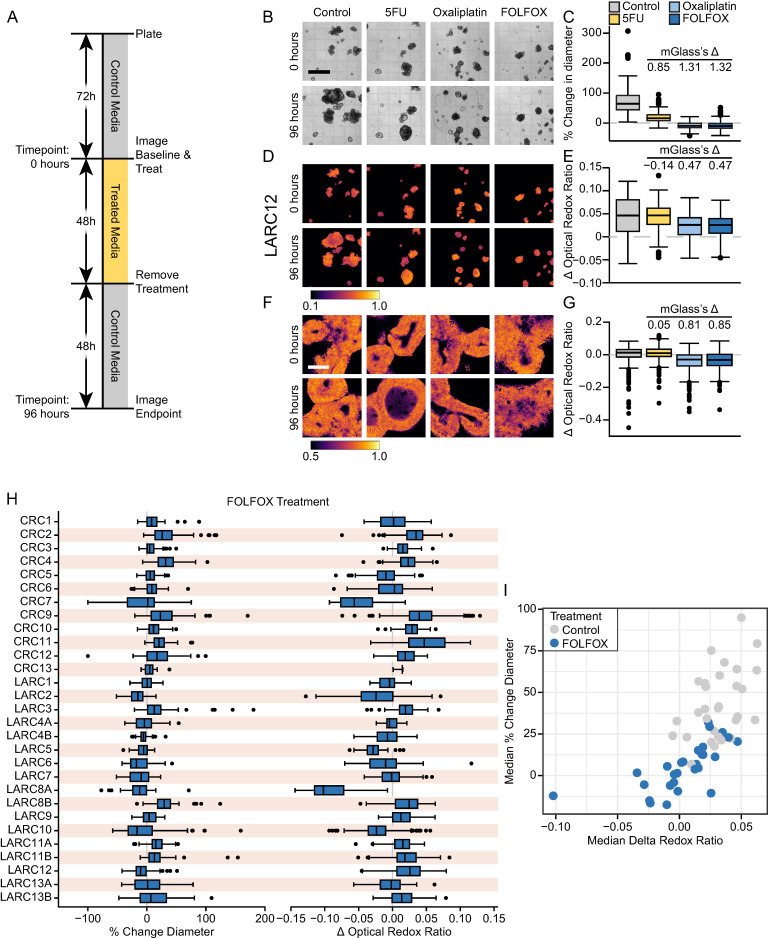
PDCOs show varying sensitivity to chemotherapy across lines. (**A**) Schema of PDCO treatment and imaging for this study. (**B**) Representative BF images of LARC12 before (Day 0) and after (Day 4) all treatments; Scale bar (black) = 1 mm. (**C**) Median relative change in diameter for LARC12 showed a significant response to FOLFOX. (**D**) Representative images of WF-ORI of LARC12 before (Day 0) and after (Day 4) all treatments. WF-ORI color bars colored according to ORR. (**E**) Median WF-ORI ΔORR for LARC12 for all treatments. (**F**) Representative images of LARC12 of 2P imaging. Scale bar = 100 µm. (**G**) Median 2P ΔORR for LARC12 for all treatments showing a slight response with FOLFOX treatment. (**H**) For all PDCO lines in both change in diameter and ΔORR, there was varying sensitivity to FOLFOX treatment across PDCOs. (**I**) All PDCO lines’ median percent change in diameter and median ΔORR when treated with FOLFOX. Blue dots indicate FOLFOX treatment, while gray indicates control.

To evaluate the utility of PDCOs to predict response to chemotherapy and XRT, the PDCOs were treated with clinically relevant treatment regimens for 96 h (Figure 2A). More specifically, CRC PDCO lines were plated 72 h prior to treatment with 5FU, oxaliplatin and the combination (FOLFOX) or 5FU in combination with the active metabolite of irinotecan, SN38 (FOLFIRI), according to the treatment received by the corresponding patient. For LARC samples, neoadjuvant chemoradiation consisting of the combination of 5FU with XRT is a standard-of-care treatment along with neoadjuvant chemotherapy [28]. LARC PDCO samples also received XRT alone and in combination with 5FU. The response to treatment was measured as relative change in the longest diameter over 96 h and change in the optical redox ratio (ΔORR).

Thirteen CRC PDCO samples from thirteen patients were treated with FOLFOX. Seventeen LARC PDCOs from thirteen patients (some patients had multiple tumor sites collected) were treated with the combination of 5FU and XRT, along with the respective single agents/modalities (Figure 2 and Supplemental Figures S1 and S2). mGlass’s Δ of relative change in diameter and ΔORR were used to quantify the drug response using prior validated thresholds for FOLFOX and FOLFIRI treated lines [4,13]. Without prior data for XRT treatments, novel thresholds were chosen based on the PDCO response herein. For XRT and the combination of XRT with 5FU, the change in diameter mGlass’s Δ threshold > 1 was evaluated as sensitive, and <0.5 was considered resistant, while ΔORR mGlass’s Δ threshold > 1 was evaluated as sensitive and <0 was considered resistant.

PDCOs showed varying sensitivities to chemoradiation not only between lines but also within the same line. An example of this variation is seen in LARC12, which was sensitive to FOLFOX treatment (median percent relative change in diameter, control: 64.0% versus (vs.) FOLFOX: −10.6%) with a mGlass’s Δ of 1.32 (Figure 2B,C). This differential sensitivity was observed in metabolic changes with WF-ORI, with a slight decrease in ΔORR with FOLFOX treatment (median ΔORR; control: 0.046 vs. FOLFOX: 0.026; mGlass’s Δ: 0.47). Two-photon (2P) imaging also showed a decrease in ΔORR for LARC12 for FOLFOX treatment with a mGlass’s Δ of 0.85 (Figure 2F,G). Further examples of differential responses across PDCO lines are shown in Supplemental Figures S1 and S2. We have recently published that WF ORR is comparable to 2P [13] and thus continue with just WF ORR for the remainder of this manuscript.

Sensitivity to FOLFOX treatment varied between PDCO lines and in individual PDCO responses within a culture in both the change in diameter and ΔORR (Figure 2H). FOLFOX treatment resulted in increased sensitivity across many PDCO lines, evidenced by a reduced median percent relative change in diameter and ΔORR compared to the corresponding control. However, a few lines were minimally responsive to FOLFOX treatment with median change in diameter and ΔORR values similar to their untreated controls (Figure 2I).

### 3.3. Organoid-Level Methods Are More Sensitive to Detect Response in PDCOs than CellTiter-Glo or Endpoint PDCO Size

**Figure 3:**
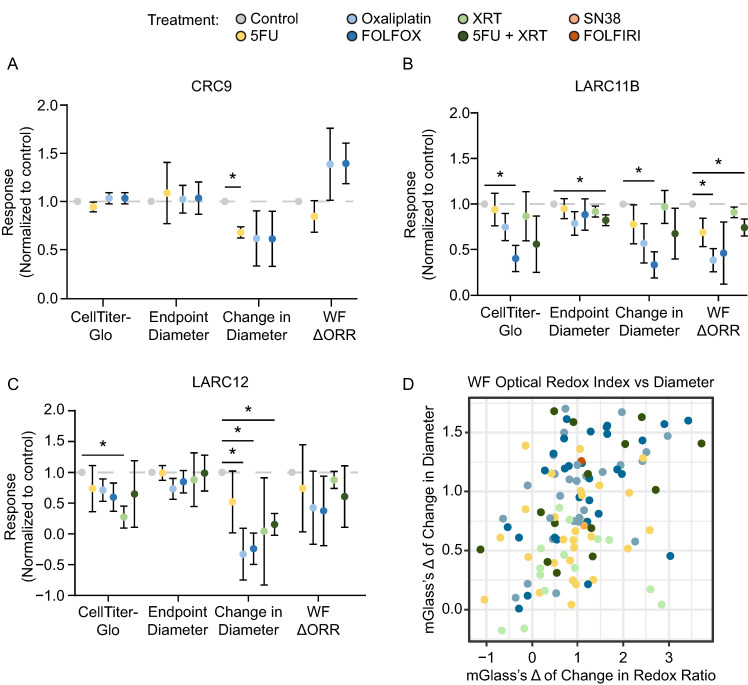
Relative change in diameter and optical redox ratio are more sensitive methods to detect response in PDCOs. (**A**) Median response was graphed for CRC9 for four methods (3D CellTiter-Glo, endpoint (Day 4) diameter, change in diameter, and ΔORR after chemotherapy treatment). No methods were able to detect a response to FOLFOX treatment. (**B**) Median response was graphed for LARC11B for all four methods after chemoradiation treatment. Change in diameter and ΔORR were able to detect a response to 5FU + XRT, while CellTiter-Glo and endpoint diameter did not. (**C**) For LARC12, the median response was graphed for all four methods. A trend of response was seen in the change in diameter after chemoradiation treatment. (gray lines drawn at axis point 1). * *p* < 0.05 for (**A**–**C**). (**D**) All mGlass’s Δ for treatments for all 26 lines were graphed when comparing the two methods, change in diameter and change in optical redox ratio. PDCOs had a varying response to these therapies across the two analysis methods.

Since we established that PDCOs respond differentially to treatment, we wanted to compare organoid-level analysis methods to the well-level methods like 3D CellTiter-Glo and endpoint diameter, often used to assess response in PDCOs. The optimal method to assess treatment response between 3D CellTiter-Glo, endpoint diameter, change in diameter, and ΔORR over 96 h was then compared across a panel of PDCO lines (Figure 3). For CRC9, all methods except for the change in diameter showed little to no response with FOLFOX treatment compared to its untreated control. Cell Titer Glo, endpoint diameter, and relative change in diameter had no significant difference between control and FOLFOX treatments (*p* = 0.42, *p* = 0.75, *p* = 0.27, respectively; Figure 3A). WF-ORI imaging showed a trend towards an increase in ΔORR (i.e., treatment resistant) between control and FOLFOX-treated PDCOs (*p* = 0.083; Figure 3A).

For LARC11B, endpoint and ΔORR showed a significant decrease between control and 5FU + XRT (*p* = 0.035, *p* = 0.041, respectively; Figure 3B). For CellTiter-Glo and change in diameter, there was no significant difference between 5FU + XRT and untreated control (*p* = 0.13, *p* = 0.18, respectively). For LARC12, PDCO-level change in diameter could pick up sensitivities to treatment where CellTiter-Glo, endpoint diameter, and ΔORR could not (Figure 3C). Change in diameter analysis demonstrated a significant decrease between 5FU + XRT (median percent relative change in diameter; control: 64.0%, as stated above, vs. 5FU + XRT: 17.5%, *p* = 0.014; mGlass’s Δ: 0.82; Figure 3C) and FOLFOX (*p* = 0.014) compared to the untreated control.

To further compare the diameter change analysis with ΔORR, these assessments were plotted across all treatment groups (Figure 3D). While there was a general trend towards a higher mGlass’s Δ across modalities, significant variation persists, indicating that these assessment methods could be complementary.

### 3.4. PDCOs Can Predict Patient Response to Chemotherapy at CRC Recurrence

**Figure 4:**
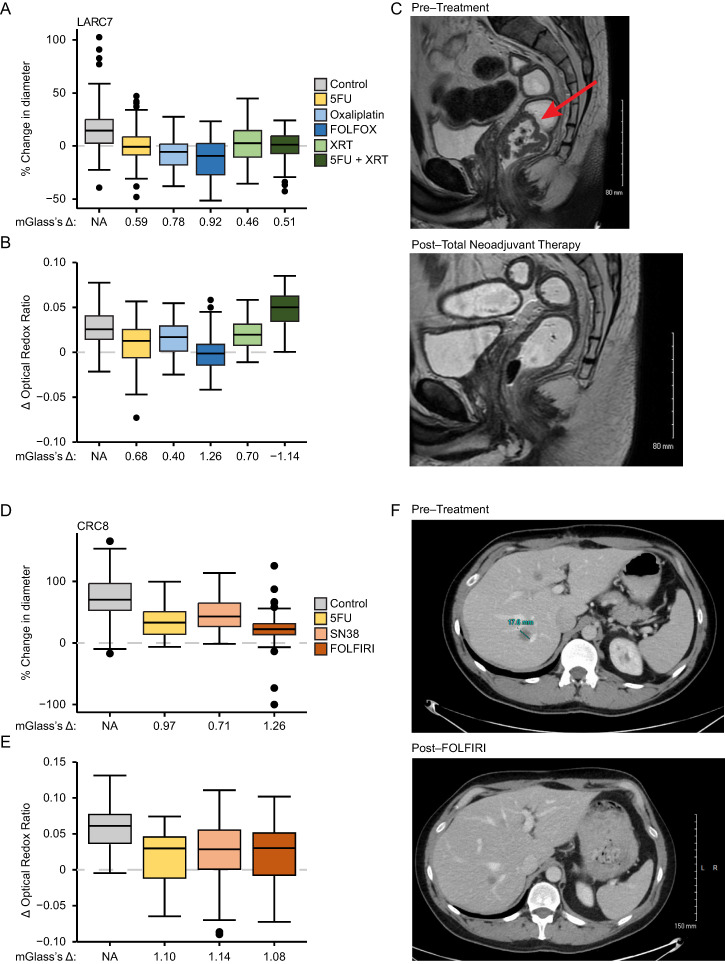
PDCO response correlates to the patient’s clinical response in both the primary and metastatic setting. (**A**) Median relative change in diameter was determined for LARC7 for all treatments, with some response seen with FOLFOX treatment. (**B**) Median of LARC7 ΔORR was determined for each treatment condition, with some response seen with FOLFOX treatment. (**C**) CT scans of the patient’s tumor before and after treatment, with the red arrow pointing to the tumor. (**D**) CRC8 median relative change in diameter was graphed for all treatment groups with a significant mGlass’s Δ after FOLFIRI treatment. (**E**) Median ΔORR of CRC8 was determined for each treatment condition, with some response seen with FOLFIRI treatment. (**F**) CT scans of CRC8 pre and post FOLFIRI treatment, with the green line marking the tumor in the liver. NA = not applicable.

We next wanted to determine if PDCOs can predict response at both initial diagnosis and recurrence. We developed PDCO lines from a LARC patient with samples collected at both diagnosis (LARC7) and recurrence (CRC8). This patient was treated initially after diagnosis with FOLFOX and subsequently received FOLFIRI after recurrence. LARC7 responded moderately to FOLFOX treatment (median percent relative change in diameter; control: 14.5% vs. FOLFOX: −9.4%) with a diameter mGlass’s Δ of 0.92 and ΔORR mGlass’s Δ of 1.26 (Figure 4A,B). Corresponding to the PDCO response, there was a 31% reduction in the sum of longest diameter (SLD) for the patient’s tumor to FOLFOX treatment, considered a partial response (PR) by RECIST v1.1 guidelines (Figure 4C).

This same patient presented with metastasis to the liver three years after FOLFOX treatment. Samples were collected during the subsequent surgery and the PDCO line, CRC8, was established as mentioned above. CRC8 was treated with 5FU, SN38, and FOLFIRI according to the patient’s clinical treatment regimen. After treatment and imaging, CRC8 had a significant response to FOLFIRI (median percent relative change in diameter; control: 70.3% vs. FOLFIRI: 22.4%) with a diameter mGlass’s Δ of 1.26 and ΔORR mGlass’s Δ of 1.08 (Figure 4D,E). Based on these results, we predicted that the patient would have a partial response to FOLFIRI treatment, and analysis of CT scans from this patient before and after treatment indicated a 48% reduction in SLD, considered a PR by RECIST v1.1 guidelines (Figure 4F).

### 3.5. PDCO Response Correlates with Patient Clinical Response Across Chemotherapy and Chemoradiation Regimens

**Figure 5:**
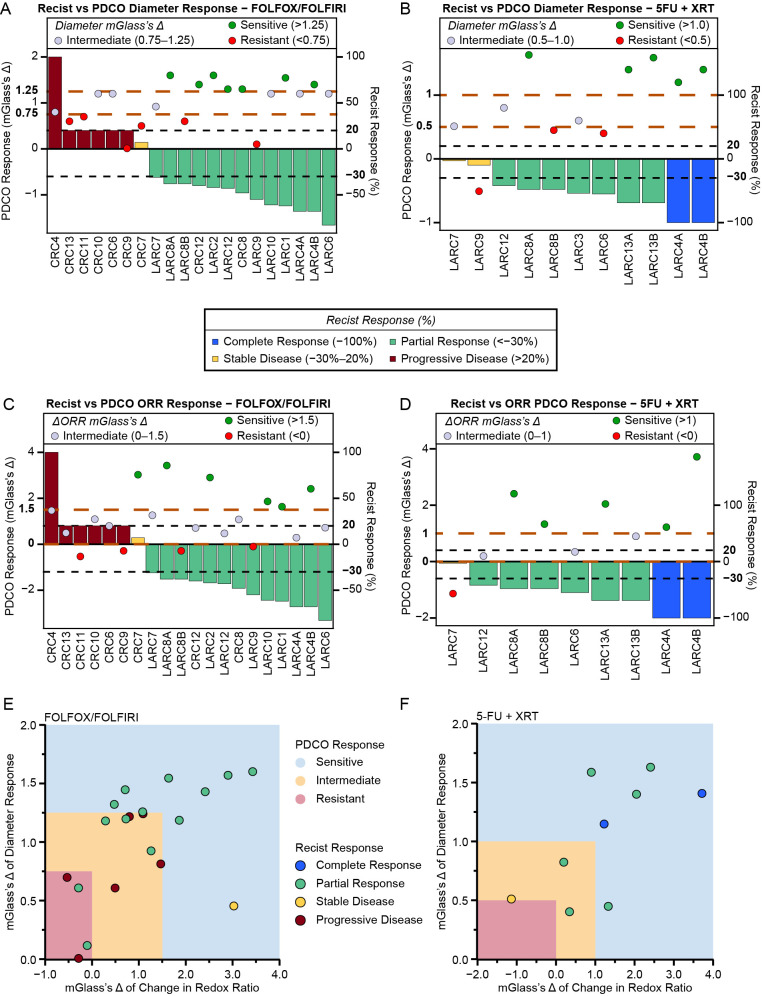
PDCO response correlates with patients’ clinical response. (**A**) mGlass’s Δ for the change in diameter of all LARC and CRC lines that were treated with FOLFOX or FOLFIRI. Bars signify RECIST 1.1 response for the patient from which the PDCOs were derived. Red indicates a patient who had progressive disease (PD), yellow indicates stable disease (SD), and green indicates partial response (PR). Orange dashed lines outline the cutoffs of the mGlass’s Δ, and black dashed lines show the cutoffs for RECIST 1.1 response. Green colored dots represent the PDCOs predicting a partial response, whereas red represents progressive disease, and gray represents intermediate. (**B**) Glass’s Δ for the change in diameter of all LARC and CRC lines that were treated with 5FU + XRT. Bars signify RECIST 1.1 response for the patient from which the PDCOs were derived. (**C**) mGlass’s Δ data for ΔORR of all LARC and CRC lines that were treated with FOLFOX or FOLFIRI. (**D**) mGlass’s Δ for ΔORR of all LARC and CRC lines that were treated with 5FU + XRT. (**E**) Comparison of clinical response with the PDCO mGlass’s Δ for data for both diameter and redox ratio responses for those PDCOs/patients treated with FOLFOX and FOLFIRI. (**F**) Comparison of clinical response with the PDCO mGlass’s Δ data for both the diameter and redox ratio responses for these PDCOs/patients treated with 5FU + XRT. Dot colors signify the RECIST 1.1 response category assigned to the patient, with blue indicating complete response (CR), green indicating partial response (PR), yellow indicating stable disease (SD), and red indicating progressive disease (PD). Graph color correlates to PDCO predicted response, with blue being sensitive, yellow intermediate, and red resistant.

To evaluate the potential of PDCOs to predict clinical response, the individual patient clinical response based on RECIST v1.1 was compared to the PDCO diameter response for FOLFOX/FOLFIRI (Figure 5A) and 5FU + XRT (Figure 5B) treatments. Twenty subjects had FOLFOX/FOLFIRI clinical response data and PDCO response data available. All subjects whose PDCOs were sensitive to these treatments had a clinical response (*n* = 7; Figure 5A). From the original 20 subjects, an additional seven subjects had an intermediate PDCO response, and 57% were clinically responsive. The remaining six subjects’ PDCOs were resistant to these treatments, and only 33% of cases achieved a clinical response.

Among the LARC patients that received 5FU and XRT treatment, all patients (*n* = 11) were treatment naïve (Figure 5B). The PDCOs were sensitive to this treatment for five subjects, and all of them had clinical responses, including two with a complete clinical response (CR). Of the three patients with PDCO intermediate response, two developed a partial response, with no complete responses. Similar results to the PDCO intermediate response group were observed with the three patients who had resistant PDCO cultures.

Additionally, these same PDCO cultures underwent WF-ORI (Figure 5C,D). Across the FOLFOX/FOLFIRI and 5FU/XRT cohorts, for those patients whose PDCOs had a ΔORR response, 91% had a clinical partial or complete response. For those PDCOs with an intermediate ΔORR response, 69% had a clinical partial response with no complete responses. For those with resistant PDCO cultures, there was a 40% partial response rate and no complete responses.

Lastly, the combination of change in PDCO diameter and change in ORR was compared to the patient’s clinical responses across chemotherapy and chemoradiation (Figure 5E,F). When using the combined assessment modalities, if a subject’s PDCOs were declared sensitive (*n* = 12), then all of those patients were without progressive disease as their best clinical response. This result included 92% of patients achieving a partial or complete response and one subject having stable disease. If the patient’s PDCOs had an intermediate sensitivity, then 60% of patients had a PR/CR, and if the PDCOs were resistant, then 50% had a PR.

## 4. Discussion

There is an unmet need for a reliable cancer model that recapitulates the human disease faithfully, especially as it relates to therapeutic responses [29]. PDCOs retain the complex morphology and heterogeneity of the tumors from which they are derived and hold great promise in this regard [4,5,7]. However, the traditional methods for assessing drug response for 3D cell cultures ignore the heterogeneity within a PDCO culture, which has a major impact on therapeutic response and limits the sensitivity for response determination [4,7].

Our group has previously reported that PDCOs display considerable heterogeneity in growth rates and treatment sensitivities among individual PDCOs within a culture [4,5,7]. Additionally, organoids are difficult to plate evenly across wells and have varying baseline sizes, limiting the sensitivity of well-level determinations of treatment response [30,31]. Ideal measurements to account for this heterogeneity would be able to track individual PDCOs over time to evaluate response on an individual level. Because it is non-destructive [13,14] and image-based, optical redox imaging has a unique advantage over other metabolic assays as it can track individual organoids and measure the same organoids at multiple timepoints, and ORI has been shown to correlate with chemotherapy response [4,5,13,14,17,18]. In this study, we directly compared well-level, single-timepoint methods (i.e., CellTiter-Glo and endpoint diameter) to individual PDCO change in diameter and optical redox ratio. The well-level methods were less sensitive in detecting treatment response compared to the diameter and WF-ORI approaches with individual PDCO tracking. These methods also allow for the visualization of proportions of the cultures with varying drug sensitivity.

Using these organoid tracking methodologies, we demonstrate that those patients whose PDCOs are sensitive to chemotherapy or chemoradiation, based on mGlass’s thresholds for individual changes in diameter or in combination with the change in optical redox ratio, have an 89–100% change in clinical PR/CR. This finding is exciting, as this degree of clinical correlation was also confirmed in our prior independent cohort and has not been observed with other means of PDCO assessment [4]. Additionally, a trend for a decreased chance of clinical response was observed with decreasing sensitivity of PDCO treatments. However, there were still a significant number of patients with clinical response where resistance occurred in the setting of PDCOs ex vivo. Results were somewhat improved by combining diameter and ORR results, as ORR correctly identified a handful of patients who were responsive with intermediate diameter responses. One possible explanation for this discordance is the overall tumor heterogeneity, of which PDCOs are just a sample. This explanation is evidenced by patient LARC8. Sample LARC8B was resistant to chemotherapy, whereas the patient had a partial response to chemotherapy, and sample LARC8A, taken from a different area of the same tumor at the same time, did show a response ex vivo. Nonetheless, this finding limits the utility of this current version of the assay for clinical use to predict responses for individual patients. However, the PDCO sensitivity threshold correlating with nearly uniform clinical response is important for using these assays to develop novel therapeutics. Those drugs or drug combinations that surpass the mGlass’s Δ threshold should be prioritized for further development with the hope of improving clinical outcomes.

Our group has assessed organoid-level treatments across multiple studies [4,5,32]. Here, we evaluated a 96 h time point with removal of the chemotherapy to mimic clinical pharmacokinetics. This change seems to have improved diameter analysis for individual PDCOs, especially post-radiation, compared to the prior 48 h time point [4]. The exchange of the study drug with media for an additional two days seems to affect the optical redox ratio to some degree, and the 48 h timepoint should be further investigated in future studies instead of the 96 h time point. This finding is consistent with prior studies that observe changes in metabolism prior to a change in tumor volume [18,33]. The iterations of these assessment techniques across these modalities, treatment schemes, and imaging timing continue to improve these methodologies. Further larger investigations should combine the individual PDCO tracking 96 h diameter measurements with 48 h change in optical redox ratio measurements. In this study, mGlass’s Δ thresholds needed to be adjusted for XRT-based treatments due to the overall lower effect sizes seen for these treatments. It is anticipated that some variations in the mGlass’s Δ thresholds will be required depending on the disease type and different treatment strategies. In all cases, several cohorts will need to be assessed to validate thresholds. Future studies should also aim to evaluate this using a CLIA-certified assay that could eventually allow for therapeutic selection studies.

Overall, these studies provide further evidence that PDCO treatment response correlates with clinical response for patients when assessed at the individual PDCO level. This study aids in the validation of PDCOs as a tool to predict patient response and to evaluate novel therapeutics for clinical translation. Future work will include expanding the sample size, diversity of treatment responses, and treatment modalities.

## 5. Conclusions

PDCOs are a powerful tool for evaluating treatment response ex vivo. As the use of PDCOs expands, it is important to keep in mind that one of the major reasons PDCOs faithfully recapitulate patient tumors is their ability to capture inter- and intra-patient heterogeneity. Evaluation of treatment response is most reflective of true patient response when methods are designed to capture this heterogeneity by measuring PDCOs at an individual as opposed to whole-well level, and by measuring changes over time as opposed to endpoint methods. Further, measurement of response at multiple levels, such as change in size and metabolism, gives the most complete picture of response. Continued refinement of the techniques presented herein will allow PDCOs to become invaluable tools for patient response prediction and drug development.

## Data Availability

The original contributions presented in this study are included in the article/Supplementary Materials. Further inquiries can be directed to the corresponding author.

## Supplementary Materials

The following supporting information can be downloaded at: https://www.mdpi.com/article/10.3390/cancers18101587/s1, Figure S1: CRC PDCO lines have differential responses to chemotherapy treatment. (A) Representative brightfield images (BF) of CRC12 before (Day 0) and after (Day 4) all treatments. Scale bar = 1 mm. (B) Median relative change in diameter of CRC12 for all treatment groups showing a significant response to FOLFOX. Brightfield scale bars (black) = 1 mm. (C) Representative images of WF-ORI of CRC12 before (Day 0) and after (Day 4) all treatments. WF-ORI color bars colored according to optical redox ratio (ORR) (D) Median ΔORR for CRC12 for all treatments. (E) Representative images of CRC12 taken on a 2P system. Scale bar = 100 µm, colored according to ORR (F) Median ΔORR for CRC12 for all treatments. (G) Representative BF images of CRC13 before (Day 0) and after (Day 4) all treatments. Scale bar = 1 mm. (H) Median relative change in diameter of CRC13 for all treatment groups showing little to no response to treatment. (I) Representative images of WF-ORI of CRC13 before (Day 0) and after (Day 4) all treatments. (J) Median ΔORR for CRC13 for all treatment groups. (K) Representative images for CRC13 2P imaging. Scale bar = 100 µm, colored according to ORR (L) Median ΔORR for CRC13 for all treatments; Figure S2: LARC PDCO response to chemoradiation treatment. (A) Representative BF images of LARC11B before (Day 0) and after (Day 4) all treatments. Scale bar = 1 mm. (B) Median relative change in diameter of LARC11B for all treatments with a slight response to FOLFOX but not for 5FU+XRT. (C) Representative images of WF-ORI of LARC11B before (Day 0) and after (Day 4) all treatments. WF-ORI color bars colored according to optical redox ratio (ORR). (D) Median ΔORR for all treatments was determined for LARC11B. (E) Representative images of LARC11B 2P imaging of all treatment groups. Scale bar = 100 µm. (F) Median ΔORR for LARC11B for all treatments.

## References

[1] American Cancer Society Key Statistics for Colorectal Cancer. https://www.cancer.org/cancer/types/colon-rectal-cancer/about/key-statistics.html.

[2] Morris V.K., Kennedy E.B., Baxter N.N., Benson A.B., Cercek A., Cho M., Ciombor K.K., Cremolini C., Davis A., Deming D.A. (2023). Treatment of Metastatic Colorectal Cancer: ASCO Guideline. J. Clin. Oncol..

[3] Scott A.J., Kennedy E.B., Berlin J., Brown G., Chalabi M., Cho M.T., Cusnir M., Dorth J., George M., Kachnic L.A. (2024). Management of Locally Advanced Rectal Cancer: ASCO Guideline. J. Clin. Oncol..

[4] Kratz J.D., Rehman S., Johnson K.A., Gillette A.A., Sunil A., Favreau P.F., Pasch C.A., Miller D., Zarling L.C., Yeung A.H. (2025). Subclonal Response Heterogeneity to Define Cancer Organoid Therapeutic Sensitivity. Sci. Rep..

[5] Pasch C.A., Favreau P.F., Yueh A.E., Babiarz C.P., Gillette A.A., Sharick J.T., Karim M.R., Nickel K.P., DeZeeuw A.K., Sprackling C.M. (2019). Patient-Derived Cancer Organoid Cultures to Predict Sensitivity to Chemotherapy and Radiation. Clin. Cancer Res..

[6] Skala M.C., Deming D.A., Kratz J.D. (2022). Technologies to Assess Drug Response and Heterogeneity in Patient-Derived Cancer Organoids. Annu. Rev. Biomed. Eng..

[7] DeStefanis R.A., Kratz J.D., Olson A.M., Sunil A., DeZeeuw A.K., Gillette A.A., Sha G.C., Johnson K.A., Pasch C.A., Clipson L. (2022). Impact of Baseline Culture Conditions of Cancer Organoids When Determining Therapeutic Response and Tumor Heterogeneity. Sci. Rep..

[8] Deben C., De La Hoz E.C., Le Compte M., Van Schil P., Hendriks J.M.H., Lauwers P., Yogeswaran S.K., Lardon F., Pauwels P., Van Laere S. (2023). OrBITS: Label-Free and Time-Lapse Monitoring of Patient Derived Organoids for Advanced Drug Screening. Cell. Oncol..

[9] Ganesh K., Wu C., O’Rourke K.P., Szeglin B.C., Zheng Y., Sauvé C.-E.G., Adileh M., Wasserman I., Marco M.R., Kim A.S. (2019). A Rectal Cancer Organoid Platform to Study Individual Responses to Chemoradiation. Nat. Med..

[10] Le Compte M., De La Hoz E.C., Peeters S., Fortes F.R., Hermans C., Domen A., Smits E., Lardon F., Vandamme T., Lin A. (2023). Single-Organoid Analysis Reveals Clinically Relevant Treatment-Resistant and Invasive Subclones in Pancreatic Cancer. npj Precis. Oncol..

[11] (2023). Promega CellTiter-Glo 3D Cell Viability Assay Technical Manual. https://www.promega.com/-/media/files/resources/protocols/technical-manuals/101/celltiter-glo-3d-cell-viability-assay-protocol.pdf.

[12] Yang J., Shay C., Saba N.F., Teng Y. (2024). Cancer Metabolism and Carcinogenesis. Exp. Hematol. Oncol..

[13] Gillette A., Udgata S., Schmitz A.E., Stoecker J.N., Kratz J.D., Deming D.A., Skala M.C. (2025). Wide-Field Optical Redox Imaging with Leading-Edge Detection Enables Assessment of Treatment Response and Heterogeneity in Patient-Derived Cancer Organoids. Cancer Res..

[14] Georgakoudi I., Skala M.C., Quinn K.P., Stringari C., Sorrells J.E., Heikal A.A., Li L.Z., Xu H.N., You S., Walsh A.J. (2025). Consensus Guidelines for Cellular Label-Free Optical Metabolic Imaging: Ensuring Accuracy and Reproducibility in Metabolic Profiling. J. Biomed. Opt..

[15] Blacker T.S., Mann Z.F., Gale J.E., Ziegler M., Bain A.J., Szabadkai G., Duchen M.R. (2014). Separating NADH and NADPH Fluorescence in Live Cells and Tissues Using FLIM. Nat. Commun..

[16] Skala M.C., Riching K.M., Gendron-Fitzpatrick A., Eickhoff J., Eliceiri K.W., White J.G., Ramanujam N. (2007). In Vivo Multiphoton Microscopy of NADH and FAD Redox States, Fluorescence Lifetimes, and Cellular Morphology in Precancerous Epithelia. Proc. Natl. Acad. Sci. USA.

[17] Shah A.T., Demory Beckler M., Walsh A.J., Jones W.P., Pohlmann P.R., Skala M.C. (2014). Optical Metabolic Imaging of Treatment Response in Human Head and Neck Squamous Cell Carcinoma. PLoS ONE.

[18] Walsh A.J., Cook R.S., Sanders M.E., Aurisicchio L., Ciliberto G., Arteaga C.L., Skala M.C. (2014). Quantitative Optical Imaging of Primary Tumor Organoid Metabolism Predicts Drug Response in Breast Cancer. Cancer Res..

[19] Desa D.E., Amitrano M.J., Murphy W.L., Skala M.C. (2024). Optical Redox Imaging to Screen Synthetic Hydrogels for Stem Cell-Derived Cardiomyocyte Differentiation and Maturation. Biophotonics Discov..

[20] Wagner M., Weber P., Bruns T., Strauss W.S.L., Wittig R., Schneckenburger H. (2010). Light Dose Is a Limiting Factor to Maintain Cell Viability in Fluorescence Microscopy and Single Molecule Detection. Int. J. Mol. Sci..

[21] Sharick J.T., Jeffery J.J., Karim M.R., Walsh C.M., Esbona K., Cook R.S., Skala M.C. (2019). Cellular Metabolic Heterogeneity In Vivo Is Recapitulated in Tumor Organoids. Neoplasia.

[22] Stringer C., Wang T., Michaelos M., Pachitariu M. (2021). Cellpose: A Generalist Algorithm for Cellular Segmentation. Nat. Methods.

[23] Samimi K., Desa D.E., Zhang X., Pham D.L., Datta R., Skala M.C. (2025). Segmentation-Guided Photon Pooling Enables Robust Single Cell Analysis and Fast Fluorescence Lifetime Imaging Microscopy. bioRxiv.

[24] Hsu A., Samimi K., Gillette A.A., Udgata S., Schmitz A.E., Zhao W., Deming D.A., Skala M.C. (2026). An Automated Image Analysis Pipeline for Wide-Field Optical Redox Imaging of Patient-Derived Cancer Organoids. Sci. Rep..

[25] Glass G.V. (1976). Primary, Secondary, and Meta-Analysis of Research. Educ. Res..

[26] Sawilowsky S.S. (2009). New Effect Size Rules of Thumb. J. Mod. Appl. Stat. Methods.

[27] Eisenhauer E.A., Therasse P., Bogaerts J., Schwartz L.H., Sargent D., Ford R., Dancey J., Arbuck S., Gwyther S., Mooney M. (2009). New Response Evaluation Criteria in Solid Tumours: Revised RECIST Guideline (Version 1.1). Eur. J. Cancer.

[28] Benson A.B., Venook A.P., Al-Hawary M.M., Azad N., Chen Y.-J., Ciombor K.K., Cohen S., Cooper H.S., Deming D., Garrido-Laguna I. (2022). Rectal Cancer, Version 2.2022, NCCN Clinical Practice Guidelines in Oncology. J. Natl. Compr. Cancer Netw..

[29] Gunti S., Hoke A.T.K., Vu K.P., London N.R. (2021). Organoid and Spheroid Tumor Models: Techniques and Applications. Cancers.

[30] Ooft S.N., Weeber F., Dijkstra K.K., McLean C.M., Kaing S., van Werkhoven E., Schipper L., Hoes L., Vis D.J., van de Haar J. (2019). Patient-Derived Organoids Can Predict Response to Chemotherapy in Metastatic Colorectal Cancer Patients. Sci. Transl. Med..

[31] Pauli C., Hopkins B.D., Prandi D., Shaw R., Fedrizzi T., Sboner A., Sailer V., Augello M., Puca L., Rosati R. (2017). Personalized in Vitro and in Vivo Cancer Models to Guide Precision Medicine. Cancer Discov..

[32] DeStefanis R.A., Schmitz A.E., Steimle A.K., Payne S.N., Sha G.C., Olson A.M., Cornelio A., Lippert A.E.L., Kraus S.G., Johnson K.A. (2025). BCL-2 Family Inhibition Enhances MTORC1/2 Inhibition in *PIK3CA*-Mutant Colorectal Cancer. Mol. Cancer Ther..

[33] Shah A.T., Diggins K.E., Walsh A.J., Irish J.M., Skala M.C. (2015). In Vivo Autofluorescence Imaging of Tumor Heterogeneity in Response to Treatment. Neoplasia.

