# Supplementary material for: Organoid Level Assessments of Human Primary and Metastatic Colorectal Cancer-Derived Organoids Predict Response to Chemotherapy and Chemoradiation
Source: Cancers (Basel). 2026 May 13;18(10):1587. doi: 10.3390/cancers18101587 (PMC13205027; doi:10.3390/cancers18101587)
Supplement: Supplementary file 1 [file cancers-18-01587-s001.zip › cancers-4280245-supplementary.pdf]

Supplemental Figures

"Organoid Level Assessments of Human Primary and Metastatic Colorectal Cancer-Derived Organoids Predict Response to Chemotherapy and Chemoradiation"

Shirsa Udgata, Alexa E. Schmitz, Amani A. Gillette, Alexandra G. Sorenson, Jeremiah M. Riendeau, Rian Engeldinger, Jordan N. Stoecker, Alyssa K. Steimle, Katherine A. Johnson, Devan Kittelson, Alexandra Isaak, Jeremy D. Kratz, Evie Carchman, Randall Kimple, Cheri A. Pasch, Melissa C. Skala and Dustin A. Deming

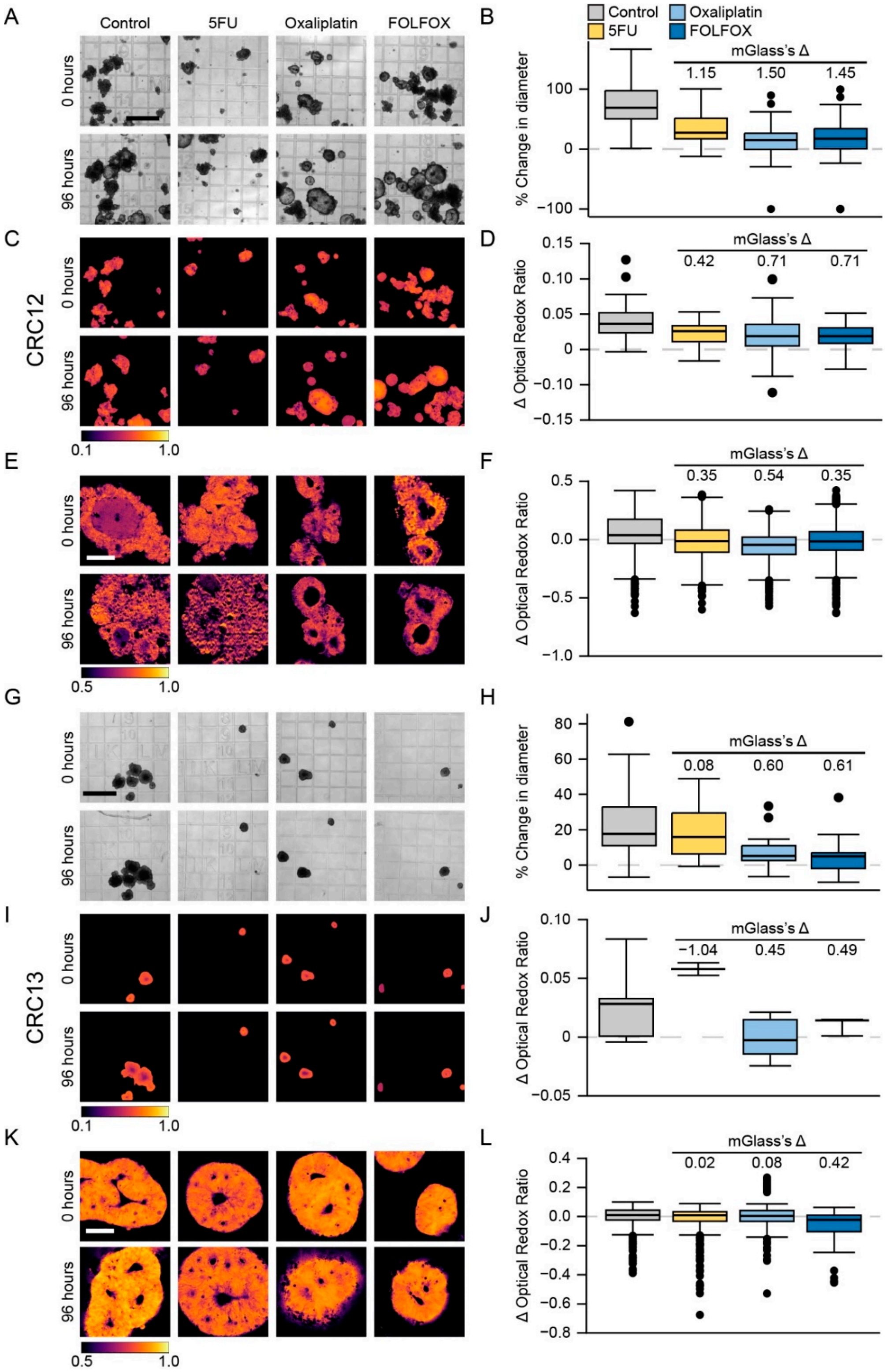

Supplemental Figure S1: CRC PDCO lines have differential responses to chemotherapy treatment. (A) Representative brightfield images (BF) of CRC12 before

(Day 0) and after (Day 4) all treatments. Scale bar = 1 mm. (B) Median relative change in diameter of CRC12 for all treatment groups showing a significant response to FOLFOX. Brightfield scale bars (black) = 1 mm. (C) Representative images of WF-ORI of CRC12 before (Day 0) and after (Day 4) all treatments. WF-ORI color bars colored according to optical redox ratio (ORR) (D) Median ΔORR for CRC12 for all treatments. (E) Representative images of CRC12 taken on a 2P system. Scale bar = 100 µm, colored according to ORR (F) Median ΔORR for CRC12 for all treatments. (G) Representative BF images of CRC13 before (Day 0) and after (Day 4) all treatments. Scale bar = 1 mm. (H) Median relative change in diameter of CRC13 for all treatment groups showing little to no response to treatment. (I) Representative images of WF-ORI of CRC13 before (Day 0) and after (Day 4) all treatments. (J) Median ΔORR for CRC13 for all treatment groups. (K) Representative images for CRC13 2P imaging. Scale bar = 100 µm, colored according to ORR (L) Median ΔORR for CRC13 for all treatments.

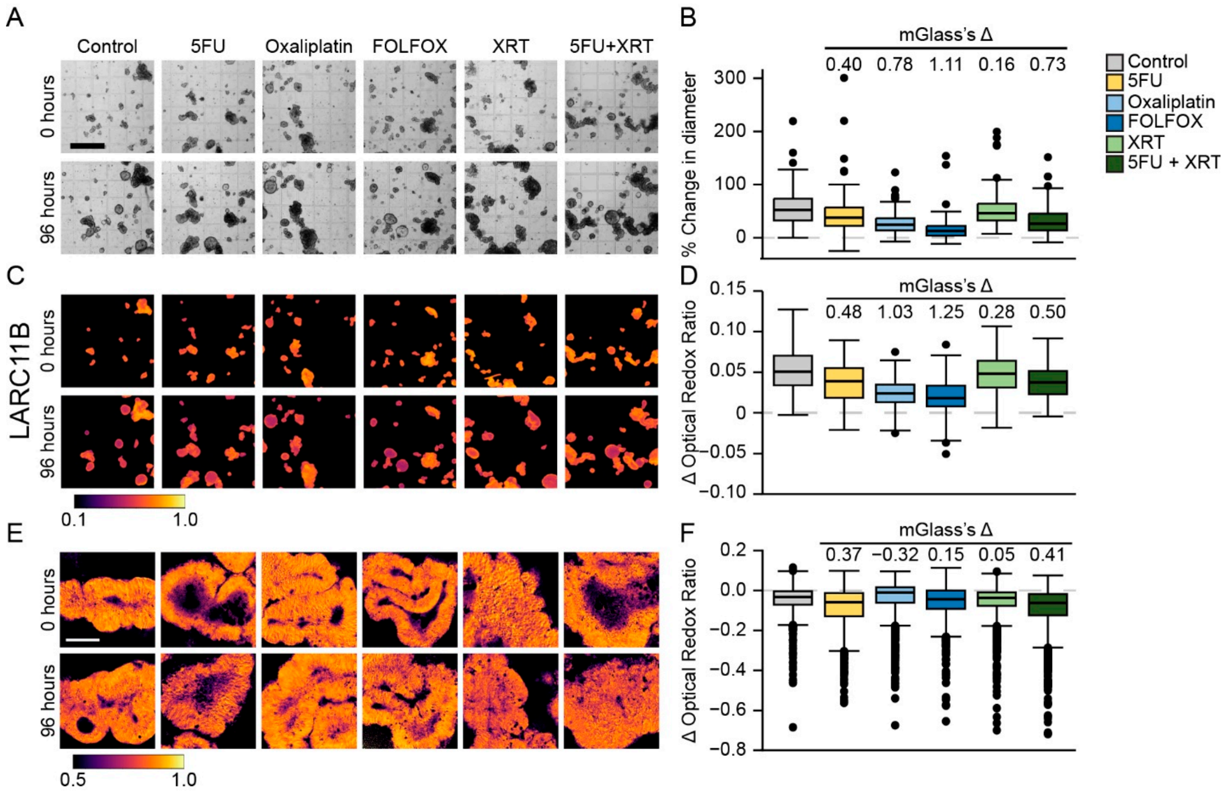

Supplemental Figure S2: LARC PDCO response to chemoradiation treatment. (A) Representative BF images of LARC11B before (Day 0) and after (Day 4) all treatments. Scale bar = 1 mm. (B) Median relative change in diameter of LARC11B for all treatments with a slight response to FOLFOX but not for 5FU+XRT. (C) Representative images of WF-ORI of LARC11B before (Day 0) and after (Day 4) all treatments. WF-ORI color bars colored according to optical redox ratio (ORR). (D) Median ΔORR for all treatments was determined for LARC11B. (E) Representative images of LARC11B 2P imaging of all treatment groups. Scale bar = 100 μm. (F) Median ΔORR for LARC11B for all treatments.
